# Maintenance Electroconvulsive Therapy Is an Essential Medical Treatment for Patients With Catatonia: A COVID-19 Related Experience

**DOI:** 10.3389/fpsyt.2021.670476

**Published:** 2021-07-15

**Authors:** Neera Ghaziuddin, Tareq Yaqub, Wael Shamseddeen, Priyanka Reddy, Hannah Reynard, Daniel Maixner

**Affiliations:** ^1^Department of Psychiatry, University of Michigan, Ann Arbor, MI, United States; ^2^Department of Psychiatry, American University, Beirut, Lebanon

**Keywords:** electroconvulsive therapy, maintenance ECT, catatonia, maintenance electroconvulsive therapy, developmental delay

## Abstract

**Aim:** Describe naturalistic clinical course over 14 weeks in a mixed adolescent and a young-adult patient group diagnosed with developmental delays and catatonia, when the frequency of maintenance electroconvulsive therapy (M-ECT) was reduced secondary to 2020 COVID-19 pandemic restrictions.

**Methods:** Participants were diagnosed with catatonia, and were receiving care in a specialized clinic. They (*n* = 9), *F* = 5, and *M* = 4, ranged in age from 16 to 21 years; ECT frequency was reduced at end of March 2020 due to institutional restrictions. Two parents/caregivers elected to discontinue ECT due to concern for COVID-19 transmission. Majority (*n* = 8) were developmentally delayed with some degree of intellectual disability (ID). Observable symptoms were rated on a three point scale during virtual visits.

**Results:** All cases experienced clinically significant decline. Worsening of motor symptoms (agitation, aggression, slowness, repetitive self-injury, stereotypies, speech deficits) emerged within the first 3 weeks, persisted over the 14 week observation period and were more frequent than neurovegetative symptoms (appetite, incontinence, sleep). Four participants deteriorated requiring rehospitalization, and 2 among these 4 needed a gastrostomy feeding tube.

**Conclusion:** Moderate and severe symptoms became apparent in all 9 cases during the observation period; medication adjustments were ineffective; resuming M-ECT at each participant's baseline schedule, usually by week 7, resulted in progressive improvement in some cases but the improvement was insufficient to prevent re-hospitalization in 4 cases. In summary, rapid deterioration was noted when M-ECT was acutely reduced in the setting of COVID-19 related restrictions.

## Introduction

The global pandemic caused by the SARS-CoV-2 (COVID-19) virus led to new challenges in medical treatment, including delivery of care to psychiatric patients (1). Electroconvulsive therapy (ECT) is administered under general anesthesia with bag and mask ventilation and is regarded as a procedure that generates aerosol with potential for disseminating viral particles. As a result, a new debate arose regarding whether or not ECT should be regarded as an essential, life-saving procedure in the context of hospitals having to reduce elective procedures to minimize the transmission of the novel coronavirus (2). Within this context, the American Psychiatric Association (APA) released multiple statements explicitly stating its position that ECT is indeed an essential and a non-elective procedure (3). Specifically, the APA clarified its concern for stopping ECT, stating, “recent studies suggest ongoing stigmatization of the severely mentally ill that may contribute to the under-utilization of necessary psychiatric interventions during a pandemic.” In example, Espinoza et al. noted inadequate understanding of ECT at the healthcare system level, skepticism among medical colleagues about the role and place of ECT in medical practice, and stigma and misperceptions which questioned whether mental illness is “real” or “serious” (2). This skepticism is concerning, given multiple studies that have repeatedly shown both the safety and efficacy of ECT across various psychiatric diagnoses across the lifespan, including in adolescents (4–8).

ECT exerts a particularly robust effect in both adults and children for the treatment of catatonia in patients with autism or intellectual disability (9, 10). There is evidence that maintenance ECT (M-ECT) defined as ECT continued beyond the index course is often necessary, safe, and may prevent relapse in catatonia which can be associated with severe sequelae such as hyperthermia, autonomic instability, electrolyte imbalances, and even death (11–14). While published literature has demonstrated the profound positive impact of acute ECT and M-ECT, there is sparse literature regarding the effects of abrupt discontinuation of M-ECT especially in individuals with catatonia and developmental delays. Even a planned discontinuation of M-ECT, exclusively studied in adults with depressive disorders or psychotic disorders, carries a substantial risk of relapse with majority experiencing relapse within 3–12 months and a frequent need to re-initate acute ECT (15, 16). On the other hand, prolonged M-ECT may provide sustained improvement without cognitive side effects (17).

The present study attempts to address the resurgence of symptoms in patients with catatonia due to the abrupt reduction or discontinuation of M-ECT secondary to the COVID-19 pandemic. Among the scant relevant literature, Tor et al. (18) describe procedures involving ECT to ensure the safety of staff and patients, while psychiatric treatment goals are met on an ongoing basis. Many programs like ours pivoted quickly during the pandemic and implemented protocols to reduce risk of infection for patients and ECT team members with COVID-19 testing at frequent intervals and thorough use of personal protective equipment (PPE).

Here, we present naturalistic outcome data, over 14-weeks, in a group of patients diagnosed with catatonia comorbid with a developmental disorder who had either experienced an abrupt cessation and/or reduction in the frequency of ECT due to the COVID-19 pandemic. The frequency of ECT was solely affected by institutional restrictions or due to personal preference of the recepients related to the pandemic. Prior to the pandemic, the frequency of ECT was individually determined and the long term goal was to continue individually titrated treatment to prevent recurrence or relapse.

## Materials and Methods

The participants were patients receiving ongoing care in a specialized clinic in the child and adolescent service at an academic center. Ethics approval for the publication of data were obtained from the Institutional Review Board (IRB). The clinical information presented in the current study was based on routine medical care, albeit modified by COVID-19 restrictions and the data were retrospectively extracted from the electronic medical records. Commonly known and observable symptoms of catatonia were monitored, which could be reliably assessed during virtual appointments by observation along with input by their caregivers. Follow up appointments were offered every 2–4 weeks, based on clinical concern and/or caregiver preference. Symptoms were monitored over 14 weeks after reducing the frequency of ECT, and compared with “baseline” observations prior to the change in ECT frequency. For the purpose of the study, Baseline 1 = symptoms noted ~4 weeks prior to change in treatment frequency; and, Baseline 2 = symptoms noted at time-point when treatment frequency was reduced. The severity of the symptoms was rated on a 3-point scale: mild = rarely experienced and/or minimal impairment; moderate = frequently experienced and/or obvious impairment in function; severe = frequently experienced and/or associated with safety concerns. The presence or absence of a symptom and its severity were assessed using all information from caregivers, by observation and other resources when available. Additionally, for the purpose of the current study, we defined maintenance ECT as individually titrated ongoing ECT administered to prevent recurrence or relapse. Of note, there are no consistent definitions of maintenance or continuations ECT which are often interchangeably used (19).

Participants were diagnosed with autism (confirmed in 6 and suspected in 3), intellectual disability (ID) in the majority (ID in 7, borderline intelligence in 1 and normal intelligence in 1); catatonia NOS, with the exception of one patient who was additionally diagnosed with Catatonia with a psychotic disorder. Catatonia NOS was recently introduced in DSM5 and is thought to improve outcome in patients diagnosed with this condition comorbid with autism spectrum disorder (ASD) and other developmental disorders; also, patients often do not have a treatable underlying psychiatric or a medical disorder (20). With the exception of one participant who was adopted and possibly subjected to maltreatment (extent unknown), remaining participants resided with one or both birth parents (6 with both birth parents and 2 with one birth parent) and did not have a history of abuse or trauma. One or both parents were employed in each household.

Other comorbid medical conditions were seizure disorders (*n* = 3; unspecified = 2, focal = 1), genetic disorders (*n* = 4; Downs = 1, KDM5C mutation = 1, 22q11.2 deletion = 1, 119 kb deletion 17q21.31= 1) and other medical disorders (*n* = 2; feeding by gastrostomy tube due to long standing poor food intake = 1, ventricular septal defect = 1). Medical workup at initial presentation included routine laboratory tests (CBC, comprehensive metabolic panel, thyroid function, and liver function tests). Additional laboratory and other tests were completed on a case by case basis and included markers for inflammation (C-reactive protein, ESR), autoimmune disorders (ANA), toxic conditions (urine toxicology, heavy metals), metabolic disorders (urine and/or serum for amino acids), and lumbar puncture with CSF examination for autoimmune encephalitis (decided in consultation with the neurology service). EEG was routinely completed at the time of the initial presentation and repeated, if suspicion for a new-onset seizure disorder emerged longitudinally. Brain MRI was completed if the patient was able to tolerate. Repeat laboratory tests, in participants who started to decline at the start of the pandemic, were completed on a case by case basis to rule out an infective, toxic or a metabolic pathology.

Medico-legal and ethical aspect were followed including consensus among 3 independent child and adolescent psychiatrists that patient should receive ECT basing their decision on treatment resistance and/or severe symptoms [institutional, state mental health code and American Academy of Child and Adolescent Psychiatry guidelines (21)] and informed consent was obtained from a parent/ legal guardian. Only one case with normal intellectual functioning was able to assent, however, a court order had to be obtained because the patient refused treatment despite parental consent.

Prior to the onset of the pandemic, individually-titrated open-ended treatment schedules were used which were determined by close follow-up in a specialty clinic for patients receiving ECT. Treatment frequency was decided using all available information from multiple sources (parental/caregiver and staff reports; catatonia rating scale was completed when possible). Additionally, prior to the pandemic, the treatment frequency was only reduced when there was evidence for *sustained* improvement over several weeks to months. This method of individually-tailored M-ECT is based on the literature, albeit limited, which supports the necessity of ECT over months or even years in patients with catatonia associated with developmental disorders (6, 11, 12).

At the time of the pandemic, due to institutional restrictions, ECT was reduced in 7 participants while family/ care-givers of two remaining participants elected to hold treatment altogether. For these 2 participants, ECT was held for the entire 14-week observation period. Treatment frequency was individually adjusted throughout the study period, depending on availability of service and decline noted in a participant (see Table 1).

**Table 1:** Emergence of Impairing Symptoms Following Reduced or Discontinued Maintenance ECT: symptom pattern over 14 weeks.

	**Baseline**	Time interval 1^*^	Time Interval 2^*^	Time Interval 3^*^
**ECT Frequency**				
2 times/week	6 (66.6%)			
3 times/week	1 (11.1%)			
Every 9 days	1 (11.1%)			
**Baseline medications**				
Benzodiazepine	7 (77%)			
Mood stabilizer	3 (33%)			
Antipsychotic	2 (22%)			
Clonidine	2 (22%)			
Memantine	1 (11%)			
Antidepressant	1 (11%)			
Change in Frequency^**^				
Increased		0 (0.0%)	4 (44.4%)	3 (33.3%)
Unchanged		0 (0.0%)	1 (11.1%)	4 (44.4%)
Reduced		7 (77.7%)	2 (22.2%)	0 (0.0%)
Stopped		2 (22.2%)	2 (22.2%)	2 (22.2%)
Motor symptoms^***^				
Agitation	2 (22.2%)	3 (33.3%)	4 (44.4%)	4 (44.4%)
Aggression	1 (11.1%)	1 (11.1%)	2 (22.2%)	1 (11.1%)
Self Injury	0 (0.0%)	0 (0.0%)	1 (11.1%)	2 (22.2%)
Motor slowness	0 (0.0%)	1 (11.1%)	2 (22.2%)	3 (33.3%)
Speech Deficit	4 (44.4%)	5 (55.5%)	7 (77.7%)	8 (88.8%)
Stereotypies	1 (11.1%)	3 (33.3%)	4 (44.4%)	4 (44.4%)
Neurovegetative symptoms^****^				
Sleep disturbance	0 (0.0%)	1 (11.1%)	3 (33.3%)	1 (11.1%)
Incontinence	0 (0.0%)	0 (0.0%)	1 (11.1%)	2 (22.2%)
Food intake	0 (0.0%)	0 (0.0%)	2 (22.2%)	2 (22.2%)
Fluid Intake	0 (0.0%)	0 (0.0%)	2 (22.2%)	1 (11.1%)

* ***Intervals:** 1= weeks 1 to 3 post treatment frequency change; Interval 2 = weeks 4 to 6; Interval 3 = weeks 7 to 14*.

** *ECT frequency is relative to the most recent frequency (e.g., change at interval 1 is compared to baseline frequency)*.

*** ***Motor symptoms**: agitation, aggression, stereotypies, slowing, self-injurious behavior, speech; symptoms reported are moderate or severe*.

**** ***Vegetative symptoms**: food intake, fluid intake, sleep, incontinence; symptoms reported are moderate or severe*.

Medications received at the onset of the pandemic included the best tolerated regimen at the time (see Table 1).

## Results

Patients (*n* = 9) were diagnosed with catatonia NOS as their primary diagnosis and had been adequately worked up, whereby the diagnosis could not be attributed to another treatable etiology. They were females (*n* = 5) and males (*n* = 4) whose ages ranged from 16 to 20 years (mean current age = 18.3 ± SD 1.6) at the time of implementing the pandemic related ECT changes. Age range at initial start of ECT was 13–20 years (mean age = 16.5 ± 2.0). The duration of ECT in months ranged from 1 to 99 (mean duration of ECT in months = 26 ± 33). Duration of catatonia diagnosis was unknown, as patients often present with the most severe forms of illness requiring ECT after having failed multiple treatments by many different providers. All patients were additionally diagnosed with intellectual disability with the exception of one participant who was also diagnosed with a psychotic disorder. Mean Baseline Bush Francis Catatonia rating score (22) prior to the start of pandemic related changes was 11.4 ± SD 3.4. ECT intervals ranged from 1 to 9 days with majority (66%) receiving 2 times per week treatment (at 3-day intervals). Symptoms at Baseline 1 and Baseline 2 were identical in each participant. At the start of the pandemic, ECT was reduced for 7 participants, while the caregivers of 2 participants decided to discontinue treatment altogether because of fear of COVID-19 infection. Participants received careful follow up during intervals 1 (observation period 1–3 weeks after the change in ECT frequency), interval 2 (weeks 4–6) and during interval 3 (weeks 7–14). Following the change or the discontinuation of ECT and during the observation period, no participant experienced a significant change in their psychosocial environment (living situation, illness or death in a family member, experience of trauma, loss of employment in care giver) or a new-onset general medical condition (infection, injury, metabolic disorder, exposure to a toxin). ECT-frequency during the 14 weeks was adjusted based on clinical determination made after the initial reduction, and included in some participants, when considered necessary, increased frequency relative to the previous interval. Therefore, the terms “increased” or “reduced” were based on comparing the frequency to the preceding observation period. Medication adjustments included dose optimization and/or addition of an agent including, lorazepam (5 cases), increased dose of clozapine (1 case), addition of memantine (3 cases), adding an alpha agonist such as clonidine to target hyperactivity and impulsivity (1 case), and cyproheptadine (to increase food intake 1 case).

Using the 3-point symptom-severity rating scale, we here report only moderate or severe symptoms based on the description in the methods section (moderate = frequent symptoms and/or associated with some impairment; severe = frequent symptoms and /or associated with safety concern for the participant). Symptom changes were evaluated within each case. Motor symptoms monitored during the study period were agitation, aggression, self-injury, motor slowing, speech deficit, stereotypies and were evaluated by observation and using parent/care-giver report. The neurovegetative symptoms were derived from parental/care-giver report and included decreased food and fluid intake, sleep problems and incontinence. Motor symptoms, relative to the neurovegetative symptoms were more prevalent during the 14-week observation period. Among the motor symptoms, most frequent were agitation, speech deficits and stereotyped motor behaviors (See Table 1 for details).

## Discussion

In a specialized patient group diagnosed with catatonia, we found a significant clinical decline over a 14-week period after ECT was either reduced or discontinued due to COVID-19 related reduced capacity in service and/or caregiver preference. Deterioration was notable within the first 3 weeks, and was progressive and persistent throughout the 14-week observation period. Medication adjustments such as adding a novel agent or maximizing the existing pharmacotherapy did not prevent the decline. Return to baseline level of function was notably slow despite resuming the baseline treatment frequency. At the baseline time-point, each participant was relatively stable and/or making progress. Four among the 9 cases in this group deteriorated sufficiently to require hospitalization over the following 6–9 month period: one participant became severely aggressive and stopped eating ultimately needing a gastrostomy-tube (G-tube) placement; a second participant was increasingly more paranoid and repeatedly eloped from home raising concern for accidental injury; a third patient became markedly more aggressive with a notable loss of speech and poor food intake which also required placement of a feeding G-tube; and, a fourth participant became progressively more agitated making it impossible for family members to care for her at home. Of note, in the first two cases among the 4 who were hospitalized, caregivers had elected to completely stop ECT due to fear of contracting COVID-19. The main effect of the pandemic was the abrupt reduction of ECT for all patients who were relatively stable but had previously not tolerated reduced schedules. The long-term goal of future treatment in each participant is to identify the best tolerated schedule and to prevent relapse.

Medication adjustments during the pandemic included either increasing the dose or adding a GABA agonist (lorazepam, zolpidem), glutamate antagonist (memantine) and/or maximizing an agent which was previously effective (alpha agonist such as clonidine; antipsychotic agent). We should note that antipsychotics were not prioritized during the medication adjustment unless the patient was already receiving this agent for psychotic symptoms. This decision was based on past history of treatment-failure with this group of agents, concern for precipitating malignant catatonia (13, 23) and a lack of clear indication such as the presence of a psychotic disorder (24).

Our findings underscore that M-ECT, as defined in the present study, is an essential service, particularly in participants diagnosed with catatonia, who may be particularly susceptible to reduced frequency or to abrupt discontinuation of ECT. The abrupt change in ECT for patients receiving M-ECT in the setting of COVID-19, however, allowed us to observe the resulting decline from either withdrawing or reducing M-ECT. Furthermore, we observed that deterioration was severe enough to require major medical interventions such as a G-tube placement in 2 among the 9 participants for life-sustaining reasons. While it is not possible to make generalizations based on a relatively small group, the degree of witnessed decline was sufficient to underscore that ECT is an essential medical service, particularly for some diagnostic groups.

There are several challenges faced by patients with catatonia and their families particularly when comorbid with developmental delays. Particularly, the diagnostic and treatment challenges involve catatonia which occurs in 12 to 17% (25, 26) of patients with autistic spectrum disorders (ASD). These include misperceptions about efficacy or the safety of high-dose benzodiazepine or ECT (27) and a lack of access to ECT due to age-related restrictions in some states in the US (prohibited in California under age 12 years; in Tennessee under 14 years, and in Texas and New York under 16 years). Additionally, lack of knowledge and experience in ECT has been noted in United States and many other countries, especially among child and adolescent psychiatrists (28–30) who often treat patients with catatonia when comorbid with developmental delays even beyond age 18 years. With the emergence of COVID-19, however, this already under-identified condition and a poorly-served patient group may have become further marginalized due to institutional regulations limiting access to ECT.

A major drawback to the use of M-ECT, irrespective of the psychiatric diagnosis, is the lack of standardization and the absence of a universally accepted definition (19). For instance, most clinicians base their practice on limited existing literature and their personal clinical experience in deciding when and how long M-ECT should be continued. In most patients, ECT is often discontinued after an initial index course when it appears to have achieved its goal of ameliorating acute symptoms. Although some present day clinicians may be less clear about the best use of M-ECT, it was first described in 1943, soon after the discovery of ECT and was used to control psychotic symptoms in schizophrenia beyond the point of initial remission (31). Historically, with the growing focus on pharmacology and psychotherapy, there was a shift away from using M-ECT in favor of alternative approaches (32). However, over time there has been a resurgence of interest in M-ECT for relapse reduction, recurrence, and rehospitalization in patients with mood disorders (32, 33) along with an evolving body of literature that has also identified the importance and safety for catatonia in pediatric and adult patients. This more recent literature involves the positive effect of M-ECT noted in catatonia patients who display uncontrollable self-injury, impulsivity, aggression, and mood lability without any evidence to suggest neuropsychological side effects (6, 9, 12, 14, 34). Wachtel (6) described ECT in 22 patients with autism and severe catatonia who had failed to respond to benzodiazepines; at the time of their report, some individual cases had received up to 700 treatments. However, a dearth of prospective and controlled data is a major drawback about the effective use of M-ECT, particularly in adolescents and young adults with catatonia comorbid with developmental delays.

Findings of this study are particularly relevant because almost nothing is known about abruptly reducing or discontinuing ECT which became a necessity during the recent pandemic. Literature pertaining to planned discontinuation of M-ECT indicates high relapse rates (15, 16), but has been exclusively reported in adults with mood or psychotic disorders while data to guide this process in younger patients including those with catatonia are lacking. Therefore, findings from the present naturalistic data suggest that discontinuing or even reducing M-ECT in catatonia, especially when done abruptly may result in serious deterioration including life-threatening symptoms such as dangerously reduced food intake, uncontrollable agitation, and/or severe impulsivity among other symptoms.

Study limitations include a small sample, reliance on parent/caregiver report, and the absence of a control group. While it is difficult to generalize results from a small number of cases, our observations illustrate the risk of emergence of acute symptoms when the frequency of M-ECT is abruptly reduced. The abrupt change in ECT frequency for patients receiving M-ECT in the setting of COVID-19 allowed us to observe increased agitation and stereotypies and worsening speech, and a more delayed appearance of reduced food intake in patients receiving M-ECT for catatonia. This report adds to a body of literature about the importance of M-ECT particularly in patients with catatonia and provides insight about the significant negative impact of COVID-19 related treatment restrictions in a special population.

## Data Availability Statement

The raw data supporting the conclusions of this article will be made available by the authors, without undue reservation.

## Ethics Statement

The studies involving human participants were reviewed and approved by IRB, University of Michigan, Ann Arbor. Written informed consent from the participants' legal guardian/next of kin was not required to participate in this study in accordance with the national legislation and the institutional requirements.

## Author Contributions

All authors have made a substantive contribution to this manuscript and have approved the final version.

## Conflict of Interest

NG receives royalties from Oxford University Press. The remaining authors declare that the research was conducted in the absence of any commercial or financial relationships that could be construed as a potential conflict of interest.
